# Proteomic Identification of Mitochondrial Targets of Arginase in Human Breast Cancer

**DOI:** 10.1371/journal.pone.0079242

**Published:** 2013-11-05

**Authors:** Rajan Singh, Nuraly K. Avliyakulov, Melissa Braga, Michael J. Haykinson, Luis Martinez, Vikash Singh, Meher Parveen, Gautam Chaudhuri, Shehla Pervin

**Affiliations:** 1 Internal Medicine, Charles Drew University of Medicine and Science, Los Angeles, California, United States of America; 2 Department of Obstetrics and Gynecology, David Geffen School of Medicine at UCLA, Los Angeles, California, United States of America; 3 Jonsson Comprehensive Cancer Center, David Geffen School of Medicine at UCLA, Los Angeles, California, United States of America; 4 Department of Biological Chemistry, David Geffen School of Medicine at UCLA, Los Angeles, California, United States of America; 5 Department of Molecular and Medical Pharmacology, David Geffen School of Medicine at UCLA, Los Angeles, California, United States of America; Rutgers - New Jersey Medical School, United States of America

## Abstract

We have previously reported arginase expression in human breast cancer cells and demonstrated that the inhibition of arginase by N^ω^ hydroxy L-arginine (NOHA) in MDA-MB-468 cells induces apoptosis. However, arginase expression and its possible molecular targets in human breast tumor samples and potential clinical implications have not been fully elucidated. Here, we demonstrate arginase expression in human breast tumor samples, and several established breast cancer cell lines, in which NOHA treatment selectively inhibits cell proliferation. The over-expression of Bcl2 in MDA-MB-468 cells abolished NOHA-induced apoptosis, suggesting that the mitochondria may be the main site of NOHA’s action. We, therefore, undertook a proteomics approach to identify key mitochondrial targets of arginase in MDA-MB-468 cells. We identified 54 non-mitochondrial and 13 mitochondrial proteins that were differentially expressed in control and NOHA treated groups. Mitochondrial serine hydroxymethyltransferase (mSHMT) was identified as one of the most promising targets of arginase. Both arginase II (Arg II) and mSHMT expressions were higher in human breast tumor tissues compared to the matched normal and there was a strong correlation between Arg II and mSHMT protein expression. MDA-MB-468 xenografts had significant upregulation of Arg II expression that preceded the induction of mSHMT expression. Small inhibitory RNA (siRNA)-mediated inhibition of Arg II in MDA-MB-468 and HCC-1806 cells led to significant inhibition of both the mSHMT gene and protein expression. As mSHMT is a key player in folate metabolism, our data provides a novel link between arginine and folate metabolism in human breast cancer, both of which are critical for tumor cell proliferation.

## Introduction

Arginine metabolism plays an important role in the regulation of tumor growth [1-3]. L-arginine is metabolized to L-ornithine and urea by arginase to provide polyamines, essential nutrients required for tumor cell proliferation [1,4]. On the other hand, L-arginine is also catabolized by the enzyme nitric-oxide synthase (NOS) to form N^ω^-hydroxy L-arginine (NOHA), an intermediate that subsequently forms nitric oxide (NO) which causes cytostasis and apoptosis of cancer cells [2,5-7]. Elevated arginase expression has been reported in tumor-associated macrophages (TAMs) that comprise up to 50% of tumor mass and foster tumor vascularization and growth [8]. Increased expression of arginase has been demonstrated to suppress NO-mediated tumor cytotoxicity and enhances tumor cell growth by providing polyamines and reducing NO production [3]. Previous studies have demonstrated high levels of serum arginase activity in human breast cancer patients compared to healthy females, levels of serum arginase activity have been positively correlated with advanced stages of breast cancer, suggesting that this enzyme might serve as a useful biomarker in breast cancer and indicator of breast cancer progression [9-11].

We have previously demonstrated elevated arginase activity in a variety of established human breast cancer cells [2]. Treatment of MDA-MB-468, a high arginase expressing breast cancer cell line with arginase inhibitor NOHA resulted in a significant inhibition of cell proliferation and induction of apoptosis [2]. This NOHA-induced apoptosis was significantly blocked in the presence of exogenous L-ornithine, suggesting that the depletion of L-ornithine or its metabolites could efficiently induce apoptosis in high arginase expressing breast cancer cells [2,7]. A detailed mechanistic analysis of the apoptotic machinery indicated that NOHA-induced apoptosis was antagonized by simultaneous treatment of the cells with exogenous L-ornithine; however, apoptotic events upstream of mitochondria such as caspase-8 induction and BH3 interacting domain death agonist (BID) cleavage remained unaltered [7]. These studies suggested that the mitochondria may be the main site of NOHA-induced apoptosis in human breast cancer cells expressing high levels of arginase. In this study, we further demonstrated the presence of arginase in a significant number of fresh human breast tumor tissues as well as in additional human breast tumor cell lines, which are sensitive to NOHA treatment. The primary objective of our study, therefore, was to identify key mitochondrial targets during NOHA-induced apoptosis in MDA-MB-468 cells that express high arginase levels. Additionally, we wanted to validate the involvement of such mitochondrial targets in clinical samples obtained from human breast cancer patients. We observed that the over-expression of Bcl2 in MDA-MB-468 cells led to a significant inhibition of NOHA-induced apoptosis, thus providing further evidence that mitochondria-mediated mechanism play an important role during the process. Using a systematic proteomics approach involving two dimensional differential gel electrophoresis (2D-DIGE) and mass-spectrometry, we identified mitochondrial serine hydroxymethyltransferase (mSHMT) as an important target of arginase in MDA-MB-468 cells. There was a significant correlation between arginase II (Arg II) and mSHMT protein as well as mRNA expression in tissue samples obtained from breast tumor patients as well as in established breast tumor cell lines. Time course examination of the induction of Arg II, and mSHMT protein and gene expression during tumor progression in nude mice injected with MDA-MB-468 cells suggested a possible correlation between these proteins. Small interfering RNA (siRNA) mediated inhibition of Arg II in MDA-MB-468 and HCC-1806 cells specifically led to significant decrease in mSHMT protein without any change in Arg I and other related proteins. Our data therefore, provides evidence that mSHMT may be a key mitochondrial target of arginase in human breast tumors and could potentially be targeted for therapeutic interventions. 

## Materials and Methods

### Cell culture

Human breast cancer cell lines MDA-MB-468, MDA-MB-231 and MDA-MB-157 (American Type Culture Collection, ATCC) were cultured in Lebovitz Medium (ATCC) containing 10% FBS, 100 IU penicillin, and 100µg/ml streptomycin (Invitrogen). Cells were cultured at 37°C. HCC 1806, HCC 70, HTB123, HCC 1937, HCC 1187, and HCC 1935 breast cancer cells (ATCC) were cultured in RPMI (Invitrogen) Medium containing 10% FBS, 100IU penicillin, and 100µg/ml streptomycin (Invitrogen). Cells were cultured at 37°C in 5% CO_2_.

### Bcl2 over-expression

 The full-length human Bcl-2 gene (kind gift from Richard J. Youle NIH, Bethesda, MD) was over-expressed in MDA-MB-468 cells as described before [6]. 

### Cell Cycle Analysis

Cell cycle analysis was performed essentially as described before (5). Briefly, cells suspended in hypotonic DNA staining buffer (0.1% sodium citrate/ .3% Triton X-100/0.01% propidium iodide/ 0.005% ribonuclease A) were incubated for 1min at 4°C and subjected to fluorescence-activated cell sorting (FACS) to analyze the percentage of cells in each phases of the cell cycle.

### Caspase Enzymatic Assay

 The Caspase-3 assay was performed as described before using 3 μg cell lysates and Ac-DEVD-AMC as a substrate [7,12]. The released AMC after specific cleavage of the substrate that becomes fluorescent was quantified using a fluorometer (Versa FluroTM, Bio-Rad) with excitation at 380 nm and emission at 440 nm [7,12]. 

## 2D-DIGE analysis and mass spectrometry

### Cell preparation and lysis

MDA-MD-468 cells (American Type Culture Collection) were cultured in DMEM medium as described before (2,6,7,12). For experimental purposes, cells were grown in 5% fetal bovine serum, allowed to seed overnight, and treated with vehicle (medium), NOHA (1mM), or NOHA plus L-Ornithine (0.5mM) for 48 hours. We previously used this concentration of NOHA to selectively inhibit cell proliferation and induce apoptosis in MDA-MB-468 cells [2,7]. Cells were washed with buffer containing 10mM Tris-Hcl, pH 7.0, and 100 mM sucrose, lysed in labeling buffer containing 7M urea, 2M thiourea, 4% CHAPS, 20mM Tris-Hcl, pH 8.8 and sonicated four times for 25 seconds with 1 minute intervals at 4°C. Protein lysates were centrifuged at 13500 x g for 15 minutes at 4°C and protein concentration was determined using 2D Quant kit (GE Healthcare, Piscataway, NJ). 

### Labeling and isoelectric focusing (IEF)

Protein samples were labeled with the *N*-hydroxysuccinimidyl ester derivates of Cy2, Cy3, and Cy5 Dyes according to the manufacture’s protocol (GE Healthcare, Piscataway, NJ) for 30 minutes on ice. One μl of 10 mM lysine solution was added to the solution and incubated for 10 minutes to quench the excess of Cy dyes. Fifty micrograms of protein from each sample were labeled with 400 pmol of Cy3 or Cy5, respectively, while the internal standard was prepared by pooling equal amounts of protein from each of the samples and then labeled at a ratio of 400 pmol of Cy2 dye per 50 μg of protein. Fifty micrograms of Cy3, Cy5 and Cy2-labeled protein samples were mixed, then up to 450 μl of rehydration solution containing 450 μl of pH 4-7 IEF buffer (7M urea, 2M thiourea, 4% CHAPS, 1% DTT, 0.5% pH 4-7 ampholytes, 5% glycerol, 10% isopropanol) was added to each sample. Samples were incubated at room temperature for 20 minutes, centrifuged at 11000 x rpm for 5 minutes at room temperature and loaded onto a pH 4-7, 24 cm Immobilized pH Gradient (IPG) strips and passively rehydrated at least for 12 h. IEF was performed using IPGPhor apparatus (GE Healthcare) with the following steps: 100V for 1 hr (hold), 200V for 1 hr (hold), 500V for 1 hr (hold), 1500V for 4 hrs (gradient), 8000V for 4 hrs (gradient), 8000V for 10 hrs (hold) for a total of 104 kVh at 20°C. 

For the basic protein separation, pH 6-9, 24 cm IPG strips were rehydrated overnight in the same rehydration solution as above except containing 2.5% of DTT and 0.75% of pH 6-11 IEF buffer. Fifty micrograms of Cy3, Cy5, and Cy2-labeled protein samples (each sample in 12 μl) were combined and then 114 μl of rehydration solution (7M urea, 2M thiourea, 4% CHAPS, 5% glycerol, 10% isopropanol) containing 2.5% DTT and 0.75% pH 6-11 ampholytes was added. Samples were incubated and centrifuged as above. Samples were applied onto rehydrated pH 6-9 strips with cup loading and IEF was performed with the following steps: at 300V for 6 hrs (hold), 1000V for 8 hrs (gradient), 6000V for 3 hrs (gradient), 8000V for 2 hrs (gradient), 8000V for 5 hrs (hold) for a total of 73 kVh at 20°C. During IEF, electrode pads soaked with milli-Q water and 2.5% DTT were changed twice. For preparative gels, equal amounts of each protein sample were pooled and 50 μgs of protein sample were labeled with Cy5 dye and 400 micrograms of unlabeled protein sample were added and the IEF was performed. Following IEF, all IPG strips were incubated in 10 ml of equilibration buffer (50 mM Tris pH 8,8, 6M urea, 30% glycerol, 2% SDS, 1% DTT, 0.002% bromophenol blue) for 15 min, followed by incubation in the alkylation buffer (50 mM Tris pH 8,8, 6M urea, 30% glycerol, 2% SDS, 4.5% iodoacetamide) for another 15 min. After, the incubation strips were loaded on 12.5% SDS-PAGE gels and run at 25V constant voltage for 1 hr, 50V for 1hr, and then 1W per gel overnight. The next morning, a 10-12W constant power per gel was applied until the bromophenol blue reached the bottom of the gel [13-15].

### Imaging analysis and mass spectrometry

After SDS gel electrophoresis, gels were scanned using the Typhoon Trio Variable Mode Imager (GE Healthcare, Piscataway, MJ) at 100 micron resolution using 488 nm laser/520BP40 filters for Cy2-labeled proteins, 532 nm laser/580BP30 filters for Cy3-labeled proteins, 633 nm laser/670BP30 filters for Cy5-labeled proteins [13-15]. Gel Images were cropped using Image Quant v.5.2 software (GE Healthcare, Piscataway, NJ). The Decyder 2D Differential Analysis v. 6.5 software suite (GE Healthcare, Piscataway, NJ) was used for identification of statistically significant differentially expressed proteins. Scanned gel images were initially analyzed using the Differential In-gel Analysis module (DIA) to create spot maps and quantify protein differences from each gel. Biological Variation Analysis module (BVA) was used to match and statistically assess all relevant protein spots across all gels. After the analysis, proteins of interests were selected based on fold change and Student’s t-test statistics. Gels were fixed, stained with Sypro Ruby (Invitrogen), scanned on the Typhoon, and re-matched to the Cy2, Cy3 and Cy5 images. Protein spots of interest were picked from gels using the Ettan Spot Picker (GE Healthcare, Piscataway, NJ) and digested with the mass spectrometry grade modified trypsin (Promega) using the ProGest robotic station (Genomic Solutions). MALDI-TOF/TOF mass spectrometry analysis was performed using Ultraflex TOF/TOF instrument (Bruker Daltonics, Bellirica, MA) as previously described [15].

### Ethical statement related to the use of human breast tumor samples

Breast tumor, and matched normal tissues were obtained from the following sources- a) Cooperative Human Tissue Network (CHTN) (http://chtn.nci.nih.gov), (tumor samples were collected using NCI funded resource under OHRP guidelines and waiver of consent) (45CFR46.101b) for anonymized samples; b) National Disease Research Interchange (NDRI) (http://ndriresourse.org) (approved biomedical research and IRB protocols from consented breast tumor patients). The research protocol was approved by the Charles Drew University Institutional Review Board (IRB) (permit number: 09-08-2229-03). 

### Quantitative Immunohistochemistry (QIHC)

Tumor and normal breast tissue sections as well as breast cancer cell lines were immune-stained using Arg I, Arg II and mSHMT antibodies and the quantitation of immuno-positive cells was performed as previously described [16-18].

### Western Blot Analysis

Tissue lysates were prepared from fresh/frozen samples and western blot analyses were conducted on 50 µg of lysates. Antibodies for Western blots include: Arg I and Arg II (Santa Cruz Biotechnology) mSHMT, VEGF and PGE2 (Abcam); ODC and Ezrin (Hybridoma bank); eNOS (BD-Transduction Laboratories); CCL18 (R&D System); GAPDH (1:5,000, Chemicon); Relative intensities of the bands were quantified by densitometric analysis (Personal Densitometer SI; Molecular Dynamics) as described previously [16-18].

### Quantitative Real-time PCR Analysis

Relative gene expression in tissue samples was analyzed by quantitative real-time PCR as previously described using following sets of primers - Arg I (forward) 5’-GGCAAGGTGGCAGAAGTC-3’ Arg I (reverse) 5’-TGGTGG TCAGTGGAGTGTTG-3’, (163 bp); Arg II (forward) 5’-CTATCAGCACTGGATCTTGTTG-3’, Arg II (reverse) 5’-GGGAGAGG AAGTTGGTCATAG-3’, (156 bp); mSHMT (forward) 5’-CTTCTGCAACCTCACGACC-3’, mSHMT (reverse) 5’-TGAGCTTATAGGGCATAGACTCG-3’ (133bp); and GAPDH (forward) 5’- TGTGGGCATCAATGGATTTGG-3’, GAPDH (forward) 5’-ACACCATGTATTCCGGGTCAAT-3’ (116bp) [16-18]. 

### Ethical statement related to animal use

This study was carried out in strict accordance with the recommendations in the Guide for the Care and Use of Laboratory Animals of the National Institutes of Health. The protocol was approved by the Institutional Animal Care and Use Committee (IACUC) on the Ethics of Animal Experiments of the Charles Drew University of Medicine and Science (permit number: I-1103-261). All surgeries were performed under isoflurane anesthesia, and all efforts were made to minimize suffering. Eight week old nude mice were purchased from Harlan Laboratories Inc. (Placentia, CA). 

### Xenotransplants

MDA-MB-468 cells (10^6^) were injected subcutaneously in nude mice and tumor growth was monitored over a period of 12-20 weeks. Measurements were obtained by caliper length and width measurements at weekly intervals for the duration of the experiment. Tumor volume was calculated as ½ (length x width^2^) [19,20]. 

### Small inhibitory RNA- mediated inhibition of Arg II

Arg II levels were down-regulated in MDA-MB-468 and HCC-1806 cells using Arg II small inhibitory RNA (siRNA) using standard techniques as before [6,17,18]. Human Arg II gene was targeted by using ON-TARGET plus SMART pool siRNA which consists of four siRNA sequences–siRNA1: 5′GUUCAAUGGCUGCGAAAGA3′, siRNA2: 5′GAUAGUGAAUCCACGCUCA3′, siRNA3: 5′CAUGAGAGAUAUUGAUCGA3′, and siRNA4: 5′GGGACUAACCUAUCGAGAA3′ (Dharmacon, Lafayette, CO, Cat# L-009454-01-0005). These siRNA were used at 100 nM concentrations with standard transfection protocol using lipofectamine 2000 (Invitrogen, Carlsbad, CA). As a control we used 100 nM random siRNA. We were able to get about ~75–80% inhibition of Arg II protein expression.

### Statistical Analysis

Data are presented as mean ± SD., and differences between groups were analyzed using the t-test or ANOVA. All comparisons were two-tailed, and *p* values <0.05 were considered statistically significant. The experiments were repeated at least three times, and data from representative experiments are shown.

## Results

### High levels of Arg II expression in human breast tumor samples and established breast cancer cell lines

**Figure 1:**
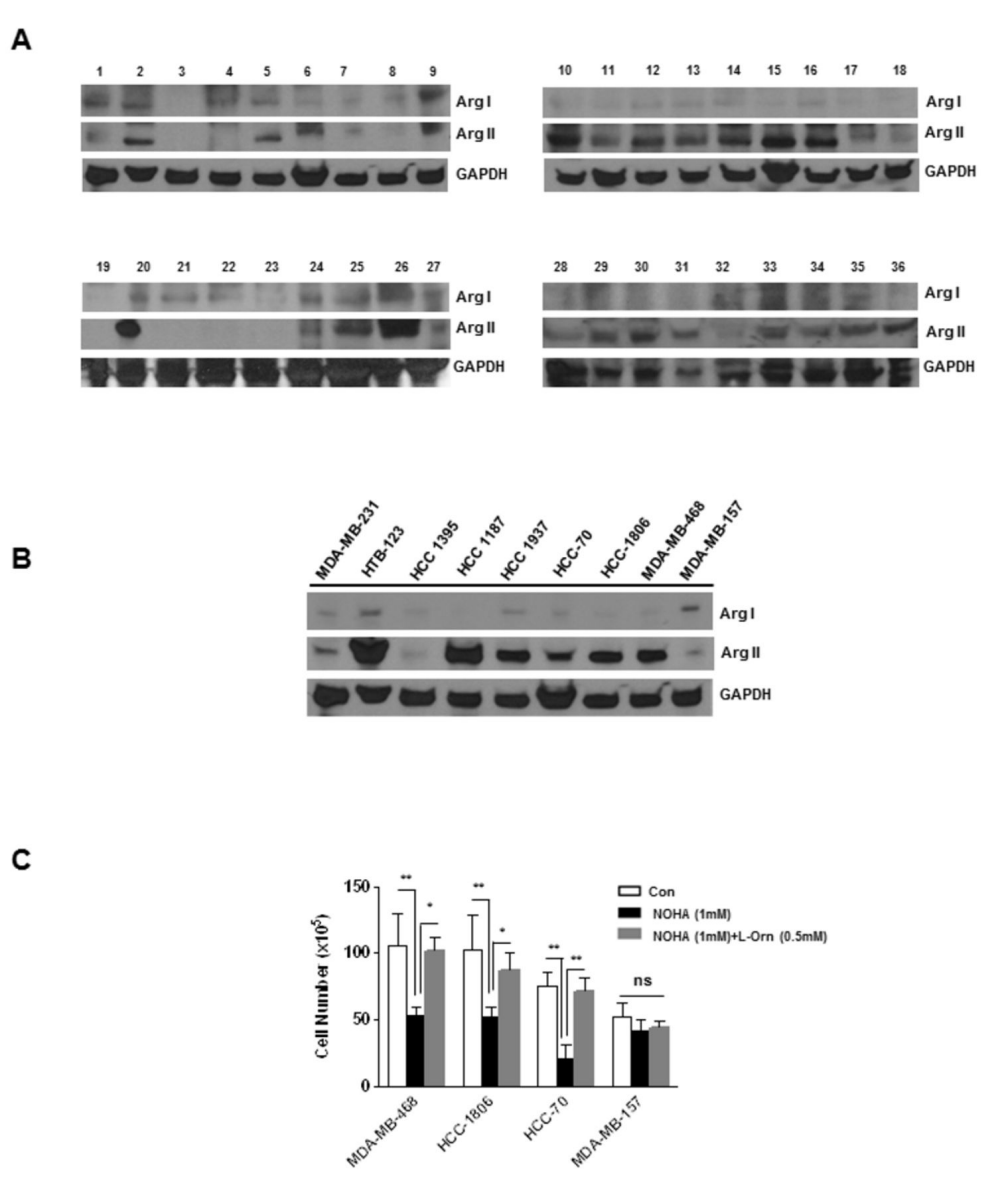
Analysis of Arg I and Arg II in human breast tumor specimen and established breast cancer cells. *A*, 50 µg of breast tumor lysates (1-36) obtained from CHTN and NDRI were analyzed by Western blot analysis using anti-Arg I or anti-Arg II antibodies. *B*, 50 µg of cell lysates obtained from established breast cancer cells were analyzed by Western blot analysis using anti-Arg I or anti-Arg II antibodies. *C*, Selective Inhibition of cell proliferation by NOHA (1mM) in high Arg II expressing breast cancer MDA-MB-468, HCC1806, HCC 70 and MDA-MB-157 cells and blockade of this effect by exogenous L-ornithine (L-Orn) (500µM). *, p≤0.05; **,p≤0.01.

We have previously demonstrated high levels of arginase expression in some selected human breast cancer cell lines, which are highly sensitive to NOHA treatment, an arginase inhibitor. In our present study, we analyze the expression of both Arg I and Arg II in human breast tumor samples obtained from various breast tumor patients as well as in some additional breast cancer cells that were not investigated before. We observed that 29 out of 36 human breast tumors expressed Arg II but only 18 out of 36 tumors expressed Arg I. The overall expression levels of Arg II was significantly higher compared to the Arg I protein expression levels (Figure 1A). Analysis of 9 human breast cancer cell lines demonstrated very high levels of Arg II expression in at least 6 cells lines (Figure 1B), including MDA-MB-468 which was previously reported to express high levels of Arg II [2]. Our current data validates our previous reports that Arg II is the predominant isoform expressed in breast cancer cells, and is also abundantly present in human breast tumor tissues obtained from both estrogen receptor positive (ER+) as well as from triple negative (TN) tumors. While Arg II was expressed in 78% of the samples, Arg I was expressed in only 47% of these samples at a lower level.

### Selective inhibition of cell proliferation in high Arg II expressing cells by NOHA

We selected three high Arg II expressing human breast cancer cell lines and one low Arg II expressing cell line, and analyzed their sensitivity to NOHA treatment after 48 hrs. All three high Arg II expressing cell lines were found to be highly sensitive to the growth inhibitory effects of NOHA (1mM).. Because of the chemical instability of NOHA at pH values above 7.0 and its extremely short half-life both *in vitro* and *in vivo* [21,22], we used high concentrations of NOHA to inhibit Arg II as reported previously by our group [2,7]. There was a significant decrease in the proliferation of MDA-MB-468 cells as reported before [2,7], which was significantly attenuated by exogenous L-ornithine treatment. Using two additional high Arg II expressing cell lines, HCC 1806 and HCC 70, we observed that these cells were also sensitive to the growth inhibitory effects of NOHA and this effect was significantly blocked in presence of exogenous L-ornithine. However, this inhibitory effect of NOHA was not observed in low Arg II expressing MDA-MB-157 cells (Figure 1C). 

### Bcl2 over-expression in MDA-MB-468 cells abolished NOHA-induced apoptosis

**Figure 2:**
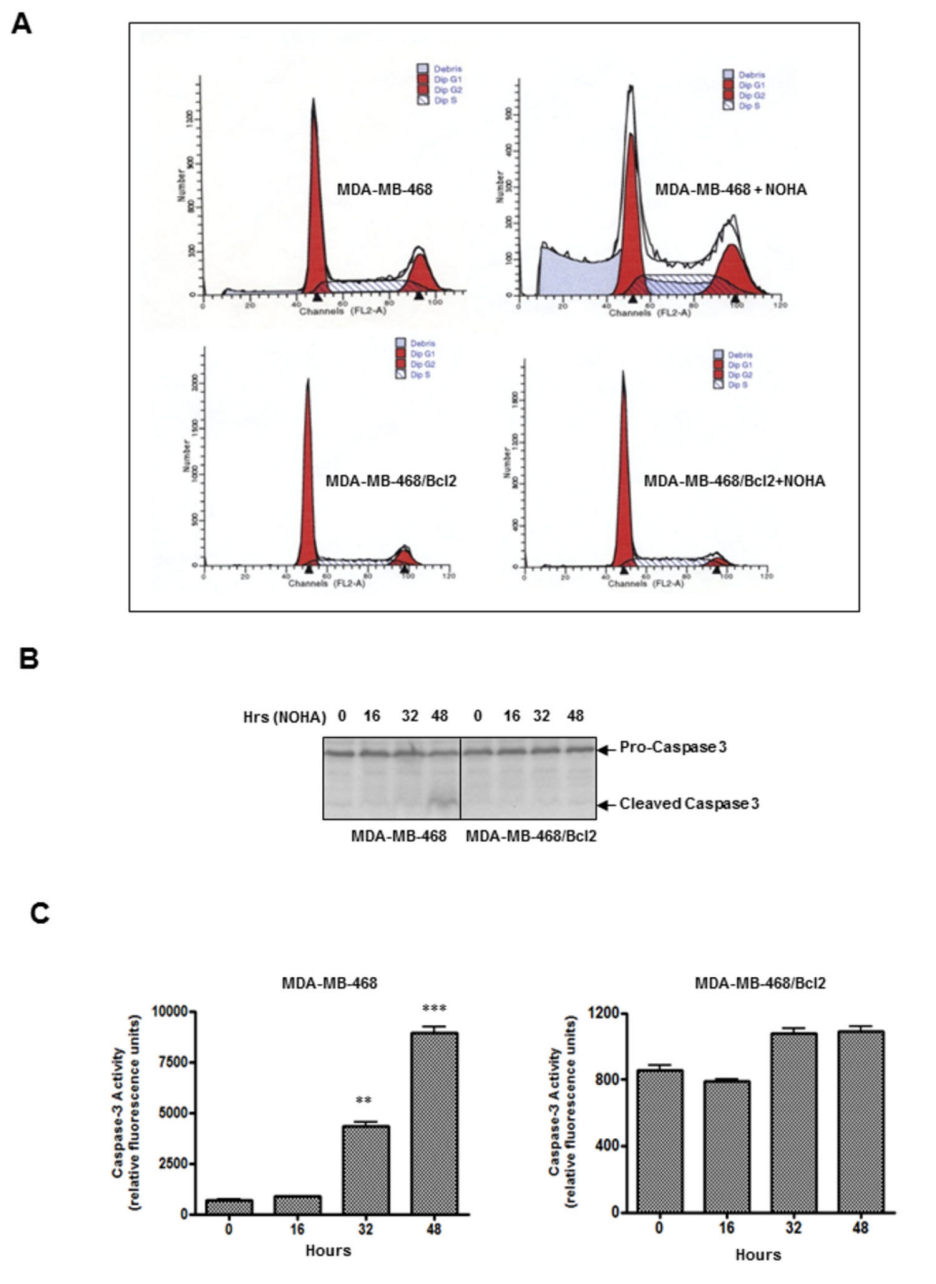
Inhibition of NOHA-induced apoptosis in Bcl2 over-expressing MDA-MB-468 (MDA-MB-468/Bcl2) cells. *A*, Control MDA-MB-468 or MDA-MB-468/Bcl2 cells were treated either with vehicle or NOHA (1mM) for 48 hrs, stained with propidium iodide and cell cycle analysis was performed. *B*, 75µg of total protein lysates were electrophoresed on 4-15% SDS/PAGE, transferred to PVDF membrane and analysis of proteolytic cleavage of caspase-3 in various treatments was performed by western blot analysis. *C*, Caspase-3 enzymatic activity in MDA-MB-468 (left panel) and MDA-MB-468/Bcl2 cells (right panel) treated with NOHA (1mM) for various time-points were analyzed. *, p≤ 0.05; **, p≤0.01.

The purpose of this experiment was to test whether the mitochondrion is the main target of NOHA-induced apoptosis in human breast cancer cells as suggested previously [7]. Since Bcl2 is a well-established anti-apoptotic protein that is known to block the release of cytochrome c from mitochondria and antagonize apoptotic process, we sought to test whether over-expression of this protein could block NOHA-induced apoptosis. We found that MDA-MB-468 cells stably expressing full-length human Bcl2 (MDA-MB-468/Bcl2) were able to antagonize the NOHA-induced increase in sub-Go population (an indicator of cell death) which occurred in control MDA-MB-468 cells (Figure 2A). We also found a significant increase in caspase-3 proteolytic cleavage (Figure 2B) and caspase-3 enzyme activities at 32 hrs (5.04±0.62 fold) and 48 hrs (11.07±1.22 fold) in control MDA-MB-468 cells after NOHA treatment as expected. However, these increases in apoptotic parameters in NOHA-treated cells were abolished in MDA-MB-468/Bcl2 cells (Figure 2B-C). Our data therefore, provides additional mechanistic insight of possible involvement of mitochondria-dependent pathways that are responsible for apoptosis induction after inhibition of arginase by NOHA in high arginase expressing MDA-MB-468 cells.

### DIGE-based proteomics profiling of NOHA-induced protein expression changes in MDA-MB-468 cells

**Figure 3:**
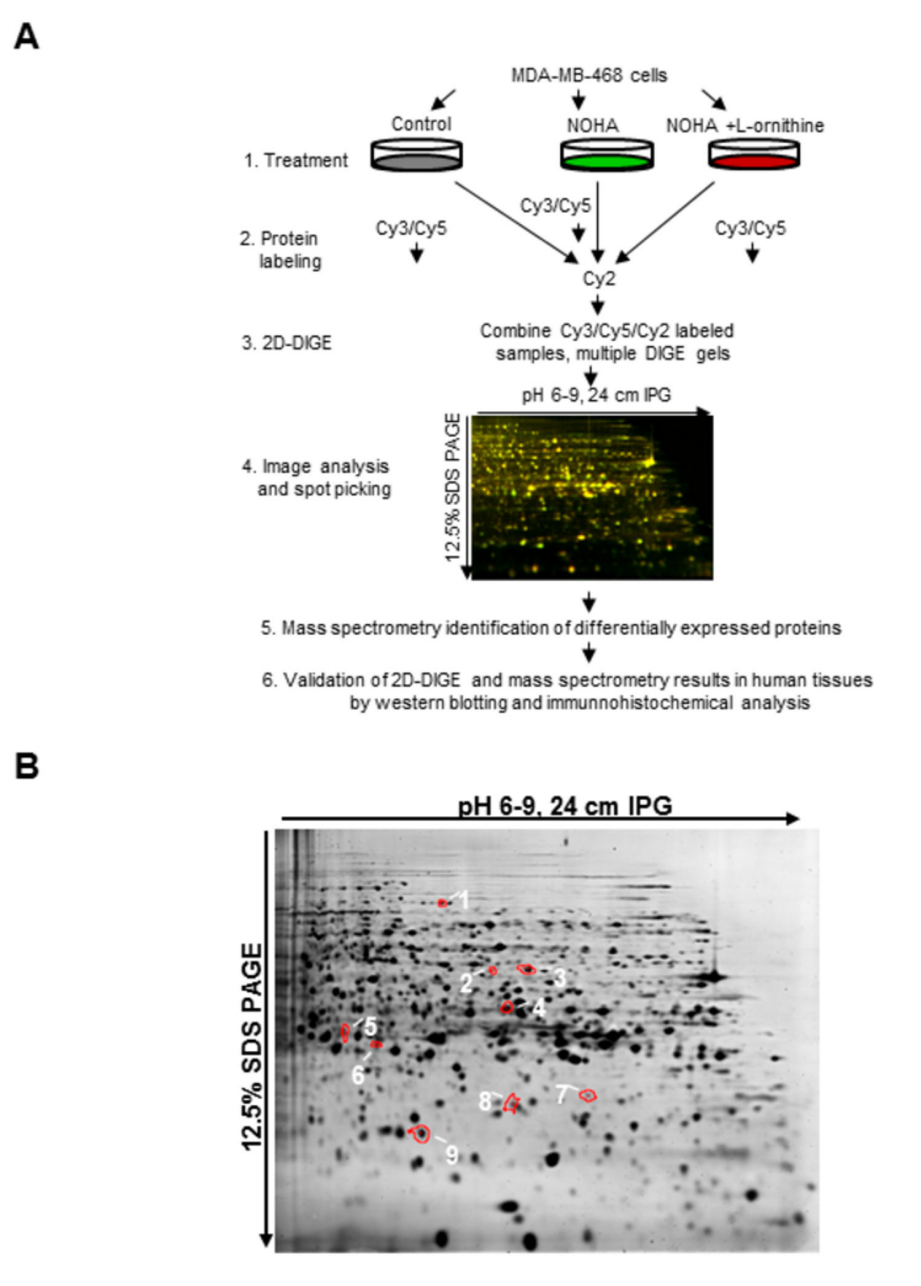
*A*, Schematic representation of 2D-DIGE/mass spectrometry analysis of MDA-MB-468 cells treated with NOHA and NOHA plus L-ornithine and validation of results in human tissues. *B*, 2D-DIGE analysis and identification of NOHA-induced mitochondrial proteins in MDA-MB-468 human breast cancer cells. Figure shows a representative Sypro Ruby-stained 2D-DIGE pH 6-9 gel image with NOHA-induced differentially expressed mitochondrial proteins. Samples labeled with Cy2, 3 and 5 dyes were cup-loaded onto a 24 cm pH 6-9 IPG strips. After isoelectric focusing, proteins were separated on 12.5% SDS PAGE. Gel images were acquired using the Typhoon scanner and analyzed by the DeCyder 2D software suite (v. 6.5). After proteins of interests were selected, picked up from gels and digested with trypsin, several differentially expressed mitochondrial proteins were identified by MALDI TOF/TOF mass spectrometry: **1** - adenylate kinase 2, **2**- ES1 protein homolog, **3**- mitochondrial 39S ribosomal proteins L28, **4** - putative mitochondrial carrier protein FLJ44862, **5** - acetyl-CoA acetyltransferase, **6** - mitochondrial 39S ribosomal proteins L39, **7** and **8** - two forms of mitochondrial serine hydroxymethyltransferase, **9** - mitochondrial aconitate hydratase. Three additional mitochondrial proteins were identified during the analysis of pH 4-7 DIGE gels: 28S ribosomal protein S22, cytochrome b-c1 complex subunit 1 mitochondrial precursor protein, and NADH-ubiquinone oxidoreductase 75 kDa subunit mitochondrial precursor protein (see Table 1).

Based on our previous reports and the effect of Bcl2 over-expression in MDA-MB-468 cells, we decided to identify differentially expressed proteins in MDA-MB-468 cells treated with either NOHA alone or in the presence of NOHA and L-ornithine using a 2D-DIGE based proteomics approach. A flow-diagram of the DIGE-based proteomics analysis and a representative gel image indicating proteins of interest is shown in Figure 3A-B. For each experimental group we used four independently grown control MDA-MB-468 cells (group 1), MDA-MB-468 cells treated with 1 mM of NOHA (group 2), or cells treated simultaneously with 1 mM of NOHA and 0.5 mM of L-ornithine (group 3) for 48 hr. Our rationale for choosing these three groups was to initially identify both mitochondrial and non-mitochondrial proteins differentially expressed in control and NOHA-treated groups. Subsequently, we wanted to identify a set of proteins in which NOHA-induced changes in mitochondrial proteins expression were antagonized in the presence of exogenous L-ornithine. Using this criteria, we detected 86 proteins in the pH 4-7 range and 36 proteins in the pH 6-9 range with changes higher than +/- 1.27-fold and with the Student’s p-value less than 0.05. We identified 46 unique differentially expressed non-mitochondrial proteins that were significantly different in NOHA-treated cells, compared to the control group (Table S1). Some of the proteins were identified in multiple spots at different locations in the gels, which are usually attributed to the presence of post-translational modifications carrying additional charges or molecular weight changes. Only one form for these multiple protein spots is listed in Table S1. In addition, 13 mitochondrial proteins were identified that were significantly different in NOHA-treated cells, compared to the control (untreated) cells (Table 1). Since in this study we focused only on differentially expressed mitochondrial proteins, we intended to identify only those specific mitochondrial proteins, for which the NOHA-induced changes were reversed by simultaneous treatment with L-ornithine. Interestingly, protein expression for all identified mitochondrial proteins was down-regulated. Although the observed protein expression changes were rather mild for most of the 13 identified mitochondrial proteins, relatively larger changes were observed with mitochondrial serine hydroxymethyltransferase (mSHMT), which was up-regulated when cells were simultaneously treated with NOHA and L-ornithine. This protein was found in more than one location on the gel (see Table 1 and Figure 3B). The identity of this protein as human mSHMT was confirmed by Mascot Peptide Mass Fingerprinting (PMF) of 15 peptides and subsequently by MS/MS peptide sequence analysis of 4 peptides as underlined in Figure S1. We subsequently focused our further analysis on this protein. 

**Table 1:** 

**Uniprotein ID**	**Protein name**	**C vs. N (fold change)**	**C vs. N (p-value)**	**C vs. N+LO (fold change)**	**C vs. N+LO (p-value)**	**MS score**	**MS2 score/seq.pep**	**pI**	**MW (kDa)**
P54819	Adenylate kinase 2, mitochondrial	-1.44	0.00073	-1.32	0.011		61/1	7.67	26689
P30042	ES1 protein homolog, mitochondrial	-1.29	0.0078	-1.2	0.086		76/2	8.5	28495
Q13084	39S ribosomal protein L28, mitochondrial	-1.55	0.012	-1.54	7.00E-05	108	158/3	8.34	30252
Q6ZT89	Putative mitochondrial carrier protein FLJ44862	-2.14	0.0067	-1.99	0.00041		29/1	8.95	33817
P82650	Mitochondrial 28S ribosomal protein S22	-1.84	0.0037	-1.67	0.0094		76/2	7.7	41425
P24752	Acetyl-CoA acetyltransferase, mitochondrial	-1.32	0.018	-1.23	0.038	103	81/3	8.98	45456
P31930	Cytochrome b-c1 complex sub 1, mitochondrial precursor	-2.22	0.0049	-2.14	0.0064	72	112/2	5.94	53297
Q9NYK5	Mitochondrial 39S ribosomal protein L39	-1.38	0.00044	-1.24	0.0012		147/2	7.56	39200
P34897	Serine hydroxymethyltransferase, mitochondrial	-2.18	0.057	-1.3	0.092	101	677/4	8.76	56414
P34897	Serine hydroxymethyltransferase, mitochondrial	-1.96	0.022	-1.41	0.025	114	71/1	8.76	56414
P10809	60 kDa heat shock protein, mitochondrial precursor	-1.42	0.0098	-1.35	0.041		105/1	5.7	61187
P28331	NADH-ubiquinone oxidoreductase 75 kDa sub, mitoch. precursor	-1.34	0.0087	-1.31	0.014	65	101/3	5.89	80443
Q99798	Aconitate hydratase, mitochondrial	-1.31	0.029	-1.24	0.085	232	118/4	7.36	86113

C: Control; N: NOHA (N-hydroxy L-arginine); LO: L-ornithine; MS: Mascot score for peptides (peptide mass fingerprinting); MS2: Mascot score for sequenced peptides; MS/MS^2^: Protein identification by MS and MS2; pI: Isoelectric point; MW: molecular weight

### NOHA-induced inhibition in mSHMT protein expression is reversed in high Arg II expressing breast cancer cells after simultaneous treatment with L-ornithine

**Figure 4:**
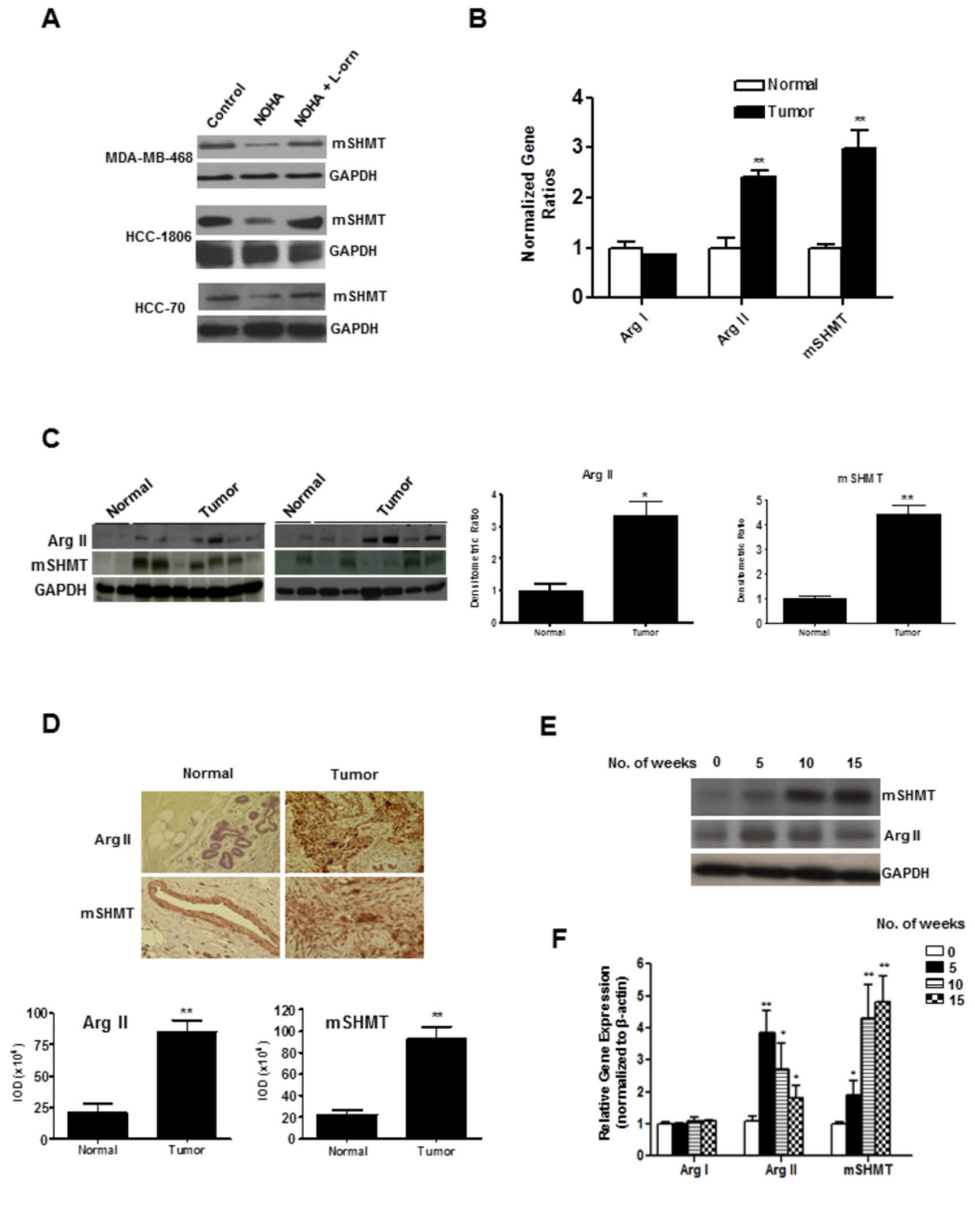
*A*, Western blot analysis of high Arg II expressing breast cancer cells MDA-MB-468, HCC 1806 and HCC 70 treated either with NOHA (1mM) alone or in combination with L-ornithine (0.5mM). *B*, Real-time quantitative PCR analysis showing selective induction of Arg II and mSHMT mRNA expression in fresh/frozen human breast tumor and matched normal tissues of Arg I, Arg II and mSHMT gene expression. *C*, Western blot analysis of Arg I, Arg II and mSHMT protein expression in normal and human breast tumor tissues (right panel) and densitometric quantitation analysis of protein expression (left panel). *D*, Top Panel, Immunohistochemical analysis of paraffin-embedded normal and breast tumor sections; Bottom Panel, Quantitative analysis of Arg II and mSHMT immuno-positive cells using ImagePro software as described in “materials and methods”. *E*, Time-course of induction of Arg II and mSHMT protein and F, gene expression in xenografts obtained from nude mice after injection of MDA-MB-468 (5x10^6^) cells *, p≤0.05; **, p≤0.01.

In order to validate our findings from the DIGE-based proteomics analysis, we analyzed the protein expression of mSHMT in MDA-MB-468 as well as in two other high Arg II expressing HCC1806 and HCC 70 cell lines treated either with NOHA (1mM) alone or in combination with L-ornithine (0.5mM) after 48 hrs. We observed that NOHA treatment led to a significant decrease in mSHMT protein expression compared to controls, but simultaneous treatment of these NOHA-treated cells with L-ornithine effectively blocked this NOHA-induced inhibition of mSHMT in all three cell lines that expressed high levels of Arg II (Figure 4A), but not in MDA-MB-157 cell line that expressed only very low levels of arginase (data not shown).

### Analysis of arginase and mSHMT gene and protein expression in human breast cancer and matched normal tissues

In order to test whether our *in vitro* identification of mSHMT as one of the key mitochondrial target of arginase in MDA-MB-468 cells has any clinical relevance, we performed quantitative real-time PCR (Figure 4B) and western blot analysis (Figure 4C, left panel) in 26 human breast tumor samples and 12 matched normal fresh tissues (Table S2) using gene specific human primer sets and respective antibodies. While individual samples were analyzed for Arg I and II as well as mSHMT gene expression, we pooled three normal tissues and two breast tumor tissues together for western blot analysis. We found a basal low level of Arg I gene and protein expression (data not shown) in both normal and tumor tissues, and there was no significant difference in gene expression between the two groups (normal vs. tumor: 1.04±0.4 vs. 0.86±0.05) (Figure 4B). On the other hand, there was significant increase in Arg II gene (2.43±0.38 fold) (Figure 4B) and protein (3.35±0.75 fold) (Figure 4C, right panel) expression in human breast tumor tissues compared to the control group. Arg II was detectable in most tumors to a varying level [11 out of 13 (2 samples pooled together) samples]. We found a very similar pattern of increase in the mSHMT gene (2.98±0.63 fold) (Figure 4B) and protein expression (4.4±0.35 fold) (Figure 4C, right panel) in tumor tissues compared to the normal tissues. The pattern of Arg II gene and protein expression in pooled tumor tissues was indicative of the overall pattern of mSHMT gene and protein expression, suggesting a relationship between the two possible candidates for breast tumor biomarkers. 

We also performed immunohistochemical staining of paraffin-embedded human breast tumor samples and matched normal breast tissue sections using anti- Arg II and anti-mSHMT antibodies (Figure 4D, top panel). We used 74 sections of breast tumor samples from patients with well-characterized clinicopathological characteristics (Table S3) and sections from 21 matched normal tissue samples to analyze the expression profiles of Arg I- , II and mSHMT. While Arg I expression was very low to undetectable in most of the normal and breast tumor tissues, Arg II immune-positive cells were detected in 13 out of 21 normal (low level) and 57 out of 74 breast tumor (intermediate to high level) sections. A comprehensive analysis of these Arg II and mSHMT immuno-positive sections by quantitative integrated optical density (IOD) (area of immune-stained cells x total intensity) suggest that Arg II levels were significantly higher in tumor tissues (4.08±0.67 fold) compared to matched normal tissues (Figure 4D, bottom panel). Expression levels of mSHMT were also found to be significantly higher (4.14±0.83 fold) in a similar set of breast tumor and matched normal sections (Figure 4D, bottom panel). In order to further investigate a possible correlation between Arg II and mSHMT expression, we generated xenografts in nude mice by injecting high Arg II expressing MDA-MB-468 cells and followed the time-course of Arg II and mSHMT expression by analyzing their protein (Figure 4E) and gene (Figure 4F) expression. Protein expression of mSHMT was found to be low initially at 5 weeks, but peaked at 15 weeks following injection (0 week: 1.0±0.11; 5 weeks: 1.9±0.8; 10 weeks: 4.30±1.8; 15 weeks: 4.8±1.4) (Figure 4E). On the other hand, relative Arg II gene expression was found to peak after 5 weeks following injection (0 week: 1.1±0.22; 5 weeks: 3.85±1.2; 10 weeks: 2.7±1.4; 15 weeks: 1.8±0.7) of MDA-MB-468 cells, thereafter, its level started to decline at a moderate rate.

### Protein expression analyses of Arg I-II, mSHMT and other related proteins in tumor specimen obtained from human breast cancer patients and established breast cancer cells

**Figure 5:**
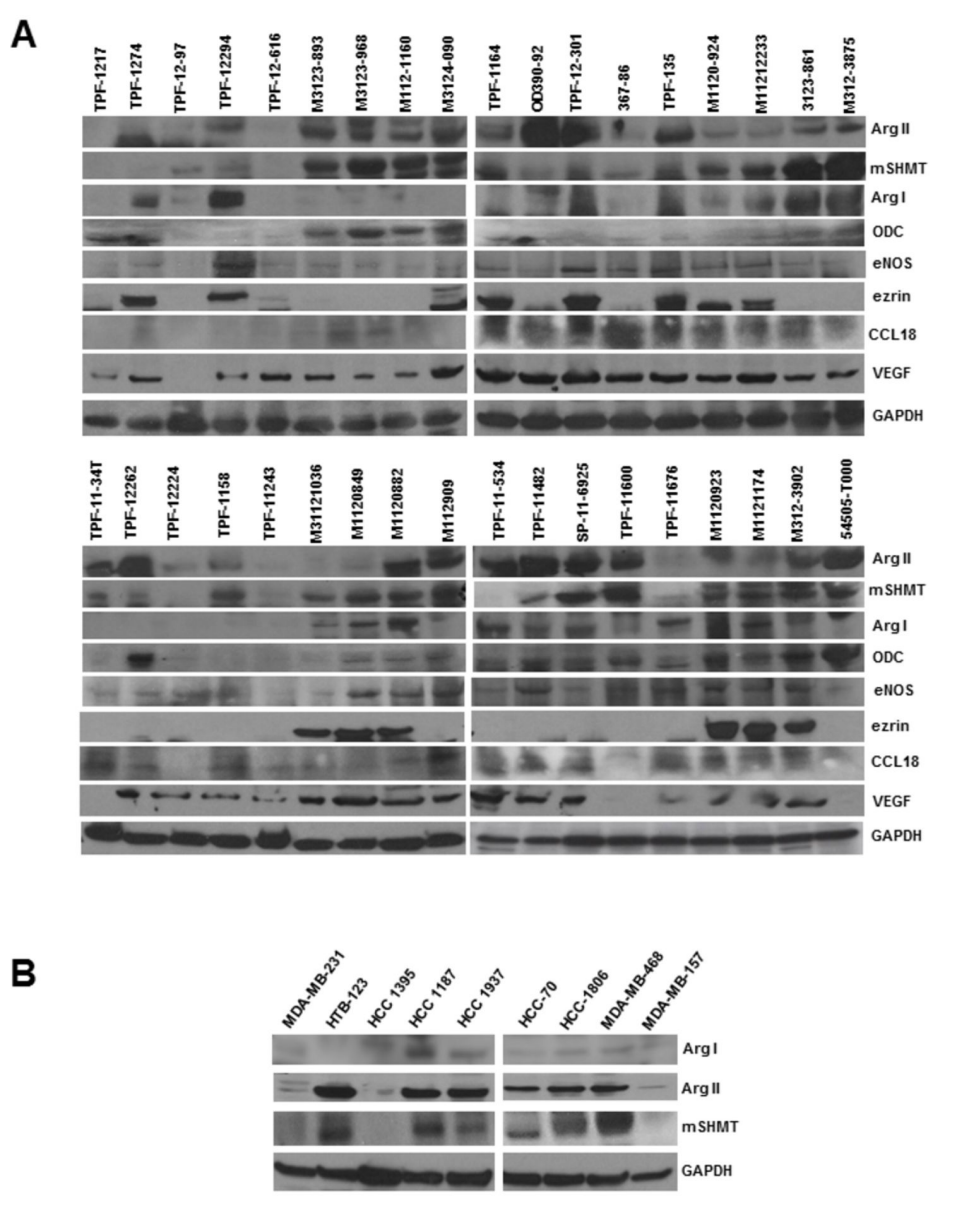
*A*, Western blot analysis of human breast tumor samples obtained from CHTN and NDRI with known pathological characteristics demonstrating a strong correlation between Arg II and mSHMT protein expression. 50 μg total tumor cell lysates were electrophoresed on PVDF membranes and analyzed by Western blot. *B*, Western blot analysis of breast cancer cells demonstrating a correlation between Arg II and mSHMT protein expression.

In order to further test the possible correlation between Arg II and mSHMT expression, we analyzed protein expression of human breast tumor specimens obtained from patients with known clinicopathological characteristics (Table S4) as well as in established human breast cancer cells. We found a very strong correlation between Arg II (and not Arg I) and mSHMT protein expression. 28 out of a total of 36 tumor samples expressed moderate to very high levels of Arg II. 26 out of these 28 Arg II positive cells expressed high to moderate levels of mSHMT expression (Figure 5A). Similarly, 6 out of 7 Arg II positive breast cancer cells expressed mSHMT (Figure 5B). On the other hand, 15 out of 17 Arg I expressing cells also expressed mSHMT. We also assessed possible correlation between mSHMT protein expression with several other proteins that are involved in arginine metabolism (eNOS), polyamine biosynthesis (ODC), and angiogenesis (VEGF). Moderate to low levels of eNOS (24 out of 36) and ODC (23 out of 36) expression were detected in these samples. VEGF was expressed in majority (32 out of 36) of these samples. We also analyzed the expression of Ezrin, a protein that was identified in our proteomics analysis as a non-mitochondrial protein that was differentially expressed between control and NOHA treated groups. We found that Ezrin was expressed in relatively smaller number of samples (14 out of 36). As high levels of Arg II are consistently detected in tumor infiltrated M2 macrophages that express CCL18, we also analyzed the expression levels of CCL18 in these tumor samples, and their correlation with Arg II and mSHMT protein expression. While 24 out of 28 Arg II expressing tissues expressed CCL18, 25 out of 26 mSHMT expressing tissues also expressed CCL18 (Figure 5A). Interestingly, 9 out of 9 triple negative (TN) human breast tumor specimens obtained from African American (AA) (7 samples) and Caucasian (2 samples) breast tumor patients expressed high levels of Arg II. The significance of this observation at this moment is not clear because of the very small sample size. However, if this finding holds true in a statistically relevant sample size, it can form a basis for novel therapeutic drug design for inhibiting arginase II or its targets for treatment of TN breast cancer, which are usually very aggressive in nature with limited therapeutic options.

### Small inhibitory RNA (siRNA)-mediated inhibition of Arg II leads to a significant decrease in mSHMT protein and gene expression

**Figure 6:**
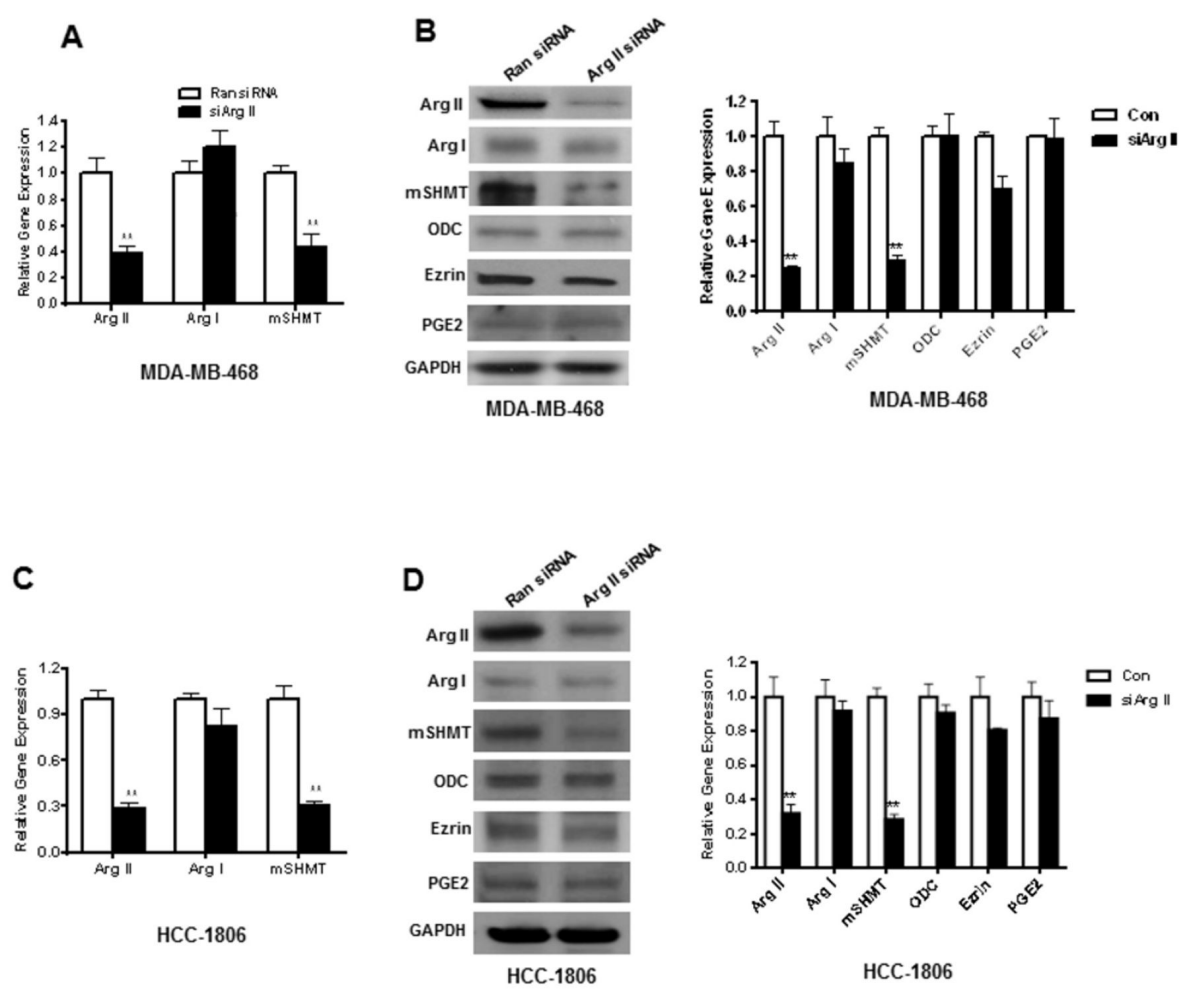
*A-B*, Selective inhibition of mSHMT gene and protein expression in MDA-MB-468 and *C-D*, HCC-1806 cells after siRNA-mediated inhibition of Arg II expression. *A*,*C*: Real-time quantitative PCR analysis using gene specific human primers (Arg I, Arg II and mSHMT) after normalization with GAPDH. *B*,*D*: Left panel, Western blot analysis; right panel, densitometric analysis of protein bands after normalization with GAPDH. Experiments were repeated three times and representative data are shown. *, p≤0.05; **, p≤0.01.

In order to further test the effect of arginase inhibition on mSHMT protein and gene expression, we performed siRNA-mediated inhibition of human Arg II in MDA-MB-468 and HCC 1806 cells. Using standard techniques, we were able to achieve significant inhibition of Arg II (61±7%) and mSHMT (56±13%) gene expression in MDA-MB-468 cells without significant change in Arg I gene expression (Figure 6A). Analysis of protein expression further confirmed significant decrease in both Arg II (76±4%) and mSHMT (71±5.5%) protein expression without any significant change in Arg I, ODC, Ezrin or PGE2 expression in MDA-MB-468 cells (Figure 6B). This selective decrease in mSHMT gene (68±9%) and protein (70±6.6%) expression following inhibition of only Arg II gene expression. This phenomenon was also confirmed in HCC 1806, the other high Arg II expressing breast cancer cell line, after siRNA-mediated inhibition of the Arg II (71.5±5%) gene, that resulted in a decrease of 72±6.5% Arg II protein expression (Figure 6C-D). Once again, there was no significant decrease in Arg I, ODC, Ezrin or PGE2 protein expression, suggesting the specificity of Arg II expression and its effect on mSHMT gene and protein expression (Figure 6C-D). This observation indicated that mSHMT might be a down-stream target of Arg II, which is mainly mitochondrial. 

## Discussion

The primary objective of this study was to identify key mitochondrial targets of arginase in MDA-MB- 468 breast cancer cell lines that express high levels of arginase. We have previously demonstrated that inhibition of arginase in MDA-MB-468 cells by NOHA induced apoptosis via Caspase-8 mediated BID cleavage, which was blocked by exogenous L-ornithine at the mitochondrial levels [7]. Furthermore, over-expression of Bcl2 in high arginase expressing MDA-MB-468 cells completely blocked NOHA-induced apoptosis. These data therefore, suggest the metabolic dependence of breast tumors on arginase pathway for their proliferation, and highlights the involvement of certain mitochondrial proteins that may be involved during the process. Accordingly, we performed proteomics analysis to identify key mitochondrial targets of arginase using NOHA as a potent arginase inhibitor. We identified two groups of proteins that were differentially expressed after treatment of MDA-MB-468 cells with NOHA. We utilized the concentration of NOHA (1mM) that was previously used by our group and others, which resulted in a significant decrease in only L-ornithine and polyamines levels in high arginase expressing cell lines. However, it had no significant effect on the cell viability in breast cancer cell lines that express only low to undetectable levels of arginase expression [2,7,23,24]. 

We identified mSHMT, a major component of folate metabolism, as the most promising protein that was differentially expressed after NOHA treatment in MDA-MB-468 cells. We also identified several non-mitochondrial proteins that were significantly altered after NOHA treatment in MDA-MB-468 cells, and for which supplementation of exogenous L-ornithine did not block NOHA-induced changes. It is possible that a significant fraction of NOHA may get metabolized to nitric oxide (NO), and nitroso-arginine [21,22] which can induce significant changes in protein expression profiles in NOHA treated cells in an arginase-independent manner. Using quantitative immunohistochemical analysis and western blot data obtained from human tumor samples, we found a significant correlation between Arg II and mSHMT expression. Furthermore, inhibition of Arg II in MDA-MB-468 as well as in HCC 1806, two high Arg II expressing cells resulted in specific inhibition of mSHMT, but not other proteins that were identified as differentially expressed non-mitochondrial proteins. Also, we did not find any significant change in the expression of cytoplasmic SHMT (cSHMT) after NOHA treatment or with siRNA inhibition of Arg II. 

The two isoenzymes mSHMT and cSHMT are involved in folate metabolism and provide active one-carbon units required for biosynthesis of nucleotides, proteins, and methyl groups by converting serine and tetrahydrofolate (THF) to glycine and methylene-THF [25-27]. While cSHMT maps to 17p11.2, mSHMT is localized at 12q13 [28]. Although the physiological functions of these two isozymes remain unclear, it is suggested that cSHMT is primarily involved in conversion of glycine to serine [28]. On the other hand, mSHMT is required for the production of glycine and N^5^, N^10^-CH2-THF [25]. Genetic variations in cSHMT and mSHMT have been associated with wide variety of human phenotypes, including childhood acute leukemia [29], rectal carcinoma [30], and prostate cancer [31]. It has been demonstrated that neoplastic tissues have significantly up-regulated levels of serine synthetic enzymes and SHMT, and the increased capacity for serine synthesis in cancer cells was coupled with its preferential utilization for the provision of nucleotide precursors for enhanced growth potential [32]. Based on their vital role in *de novo* biosynthesis of purines and thymidylate, folate-requiring enzymes have long been considered viable targets for anti-cancer therapy [33-37]. Methotrexate (MTX) is one of the most widely used anti-folate agents in chemotherapy, blocking de novo nucleotide synthesis by depleting reduced THFs, mainly through inhibition of dihydrofolate reductase (DHFR) and thymidylate synthase (TS). It displays substantial efficacy in treatment of a number of malignancies including breast cancer, head and neck cancer, non-Hodgkin lymphoma, osteosarcoma, bladder cancer and choriocarcinoma [38]. It has been postulated that increased SHMT activity is associated with the development of MTX resistance [39]. In this regard, direct inhibition of SHMT has been suggested as a promising potential target for anti-cancer therapy [32,39,40].

Identification of mSHMT as a target of arginase in human breast cancer suggests a potential link between arginine and folate metabolism; however, the mechanisms by which mSHMT expression and activity is regulated by Arg II is not known. Both of these proteins are localized in the mitochondria and are essential for enhanced polyamine and nucleotide synthesis, which are required to meet an increased demand for growth and proliferation in breast cancer cells. Methotrexate, a folate antagonist has been reported to induce cellular differentiation in colon cancer cells by depleting intracellular purine levels, an effect which was associated with a reduction in polyamine levels [41], suggesting a potential link between folate and polyamine pathways. Both isoforms of SHMT (cytoplasmic and mitochondrial) have previously been identified as bona-fide Myc-responsive genes along with others including ornithine decarboxylase (ODC) in a functional screen [42]. ODC is a major enzyme that regulates polyamine biosynthesis by converting L-ornithine; the final product of arginase catalyzed reaction, to putrescine for spermidine and subsequently to spermine biosynthesis and thereby controls cell growth and proliferation [43]. Our current findings therefore, provide a novel mechanism that may be exploited during anti-folate therapy for breast cancer treatment via depletion of the cellular pool of polyamines required to meet the high demand of growth and proliferation of tumor cells in combination with potent inhibitors of arginase [44,45]. Future studies are in progress to understand the molecular mechanisms by which inhibition of arginase may regulate both of these key metabolic pathways required to meet high proliferative demand in breast cancer cells and tissues. 

## Supporting Information

Figure S1 **Mass spectrometry identification of human mitochondrial serine hydroxymethyltransferase (mSHMT) protein.** Proteins picked from gels were digested with trypsin and analyzed by MALDI TOF-TOF mass spectrometry. Figure1 shows the results of Mascot Peptide Mass Fingerprinting (PMF) and MS/MS Ion searches. Protein sequences for the mSHMT (human mitochondrial form, NP_005403) and cSHMT (human cytosolic form, NP_004160) are aligned as previously described (33). A total of 15 peptides were matched using Mascot PMF (32% coverage), and 4 peptides were then sequenced using MS/MS peptide sequence analysis. All matched peptides are shown in red; four unique MS/MS-sequenced peptides are underlined. Asterisks on top of the matched peptides indicate amino acid sequence differences between the human mitochondrial (mSHMT) and the cytosolic hydroxymethyltransferase (cSHMT) proteins.
(TIF)

Table S1 (XLS)

Table S2 (DOCX)

Table S3 (DOCX)

Table S4 (XLS)
