# Correction: Proteomic Identification of Mitochondrial Targets of Arginase in Human Breast Cancer

**DOI:** 10.1371/annotation/7e9bf57e-a45f-4a22-95ee-eece21d282c8

**Published:** 2013-12-13

**Authors:** Rajan Singh, Nuraly K. Avliyakulov, Melissa Braga, Michael J. Haykinson, Luis Martinez, Vikash Singh, Meher Parveen, Gautam Chaudhuri, Shehla Pervin

Two errors were introduced in the preparation of this article for publication.

The title of Table 1 is missing. It should read: "Table 1: NOHA-induced changes in mitochondrial proteins in MDA-MB-468 cells."

Figure S1 was replaced by Figure 1. Please find a correct version of Figure S1 here: 
