# Supplementary material for: Proteomic Identification of Mitochondrial Targets of Arginase in Human Breast Cancer
Source: PLoS One. 2013 Nov 5;8(11):e79242. doi: 10.1371/journal.pone.0079242 (PMC3818427; doi:10.1371/journal.pone.0079242)
Supplement: Table S2 — (DOCX) [file pone.0079242.s003.docx]

|  | **Normal** | **Tumor** |
| --- | --- | --- |
| **Total Number** | 12 | 26 |
| **Ethnicity**  African American  Caucasian | 4(33.3%)  8(66.67%) | 9(34.6%)  17(64.4%) |
| **Age**  ≥50 years  ≤50 years | 9(75%)  3(25%) | 21(80.8%)  5(19.2%) |
| **Pathological Stage**  Stage 1  Stage 2  Stage 3  Stage 4 | 1(8.3%)  7(58.4%)  4(33.3%)  0(0.00%) | 4(15.4%)  11(42.3%)  9(34.6%)  2(7.7%) |
| **Lymph node**  Positive  Negative | 7(58.3%)  5(41.7%) | 18(69.2%)  8(30.8%) |
| **Histological Grade**  Ductal  Lobular  Mixed | 8(66.7%)  3(25.0%)  1(8.3%) | 17(65.4%)  3(11.5%)  5(19.1%) |
