# Supplementary material for: Proteomic Identification of Mitochondrial Targets of Arginase in Human Breast Cancer
Source: PLoS One. 2013 Nov 5;8(11):e79242. doi: 10.1371/journal.pone.0079242 (PMC3818427; doi:10.1371/journal.pone.0079242)
Supplement: Table S3 — (DOCX) [file pone.0079242.s004.docx]

|  | **Normal** | **Tumor** |
| --- | --- | --- |
| **Total Number** | 21 | 74 |
| **Ethnicity**  African American  Caucasian | 8(38.1%)  13(61.9%) | 31(41.9%)  43(58.2%) |
| **Age**  ≥50 years  ≤50 years | 16(76.2%)  5(23.8%) | 58(78.4%)  16(21.6%) |
| **Pathological Stage**  Stage 1  Stage 2  Stage 3  Stage 4 | 4(19.0%)  11(52.4%)  5(23.8%)  1(4.8%) | 17(23.0%)  34(45.9%)  19(25.7%)  4(5.4%) |
