# Supplementary figures and images for: Correction: Proteomic Identification of Mitochondrial Targets of Arginase in Human Breast Cancer
Source: PLoS One. 2013 Dec 13;8(12):10.1371/annotation/7e9bf57e-a45f-4a22-95ee-eece21d282c8. doi: 10.1371/annotation/7e9bf57e-a45f-4a22-95ee-eece21d282c8 (PMC3865333; doi:10.1371/annotation/7e9bf57e-a45f-4a22-95ee-eece21d282c8)

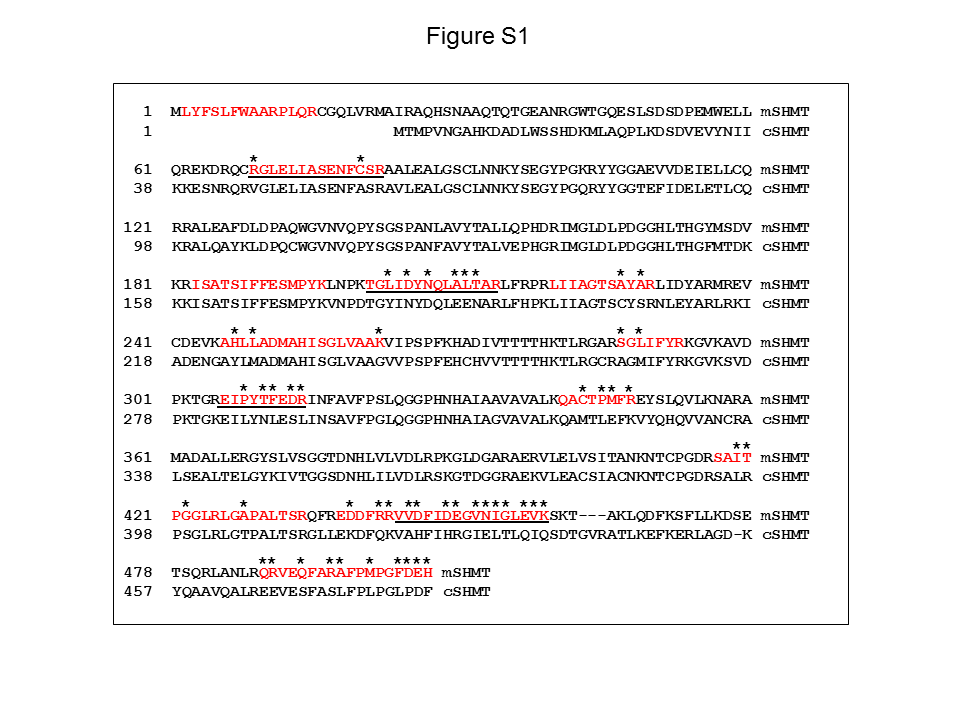

Supplement: Supplementary file 1 [file pone.7e9bf57e-a45f-4a22-95ee-eece21d282c8.s001.tif]
